# Supplementary figures and images for: DNA passes through cohesin’s hinge as well as its Smc3–kleisin interface
Source: eLife. 2022 Sep 12;11:e80310. doi: 10.7554/eLife.80310 (PMC9467508; doi:10.7554/eLife.80310)

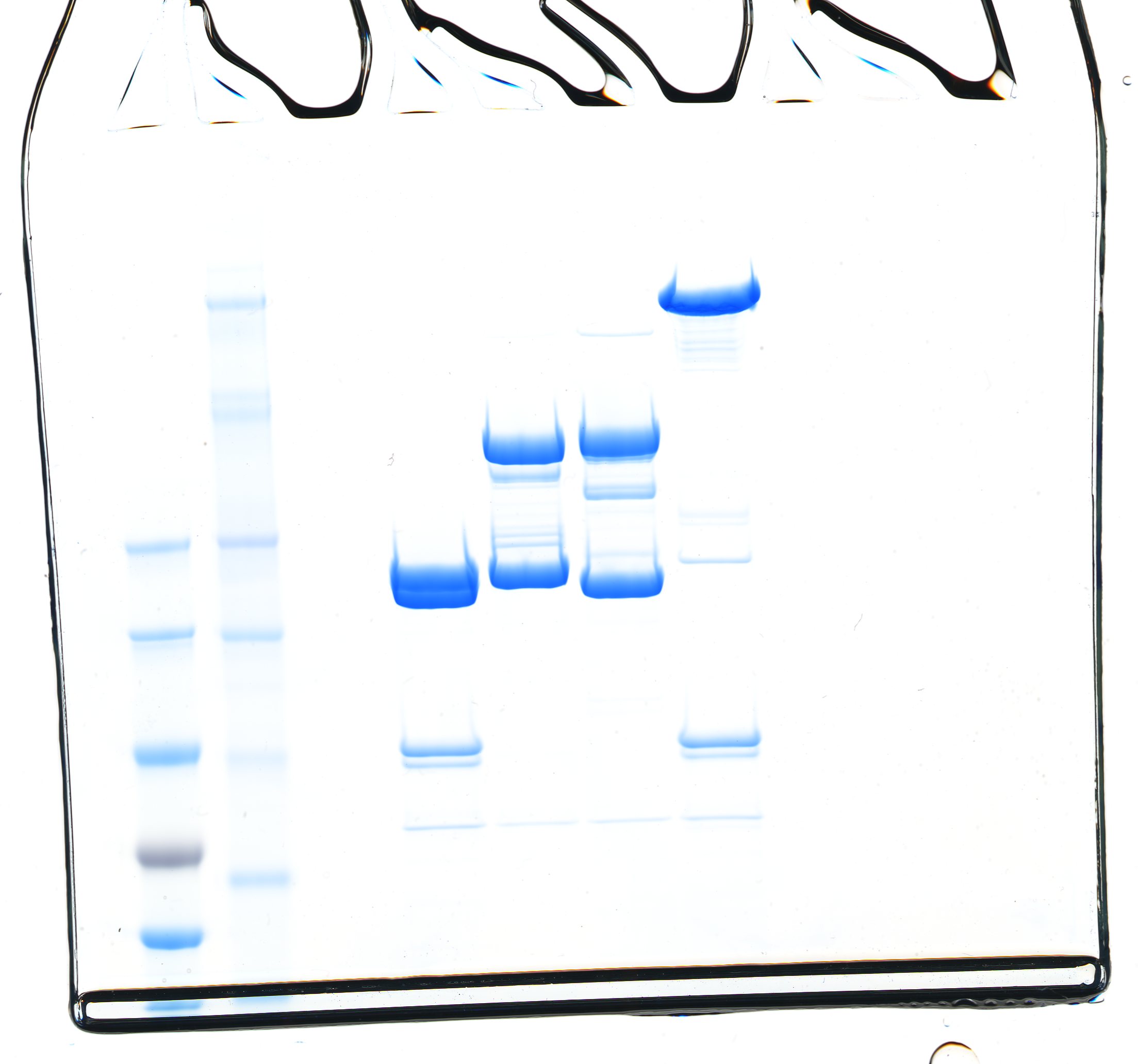

Supplement: Source data 1. [file elife-80310-data1.zip › Figure 1B - source data.jpg]

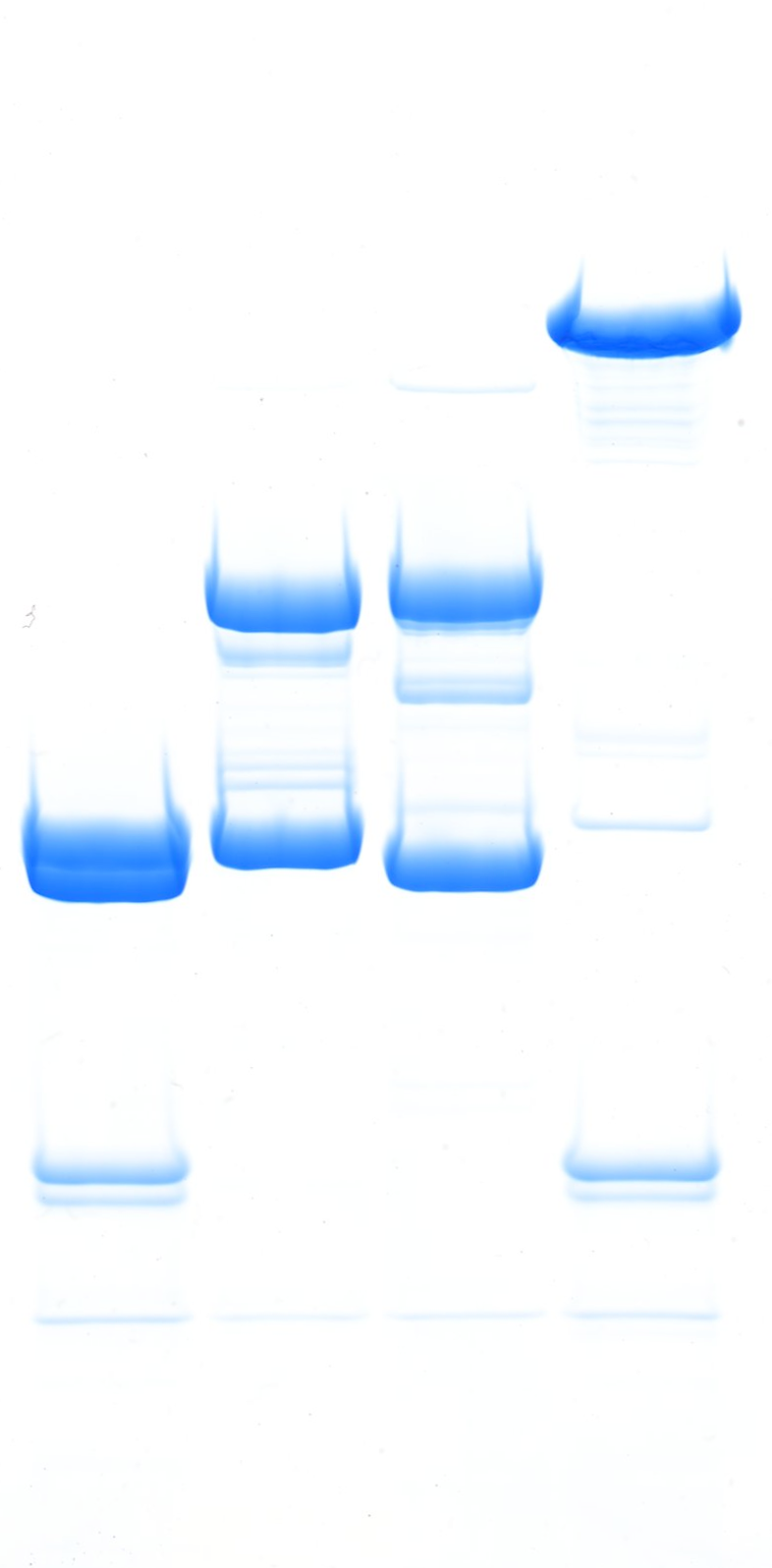

Supplement: Source data 1. [file elife-80310-data1.zip › Figure 1B - source data cropped.png]

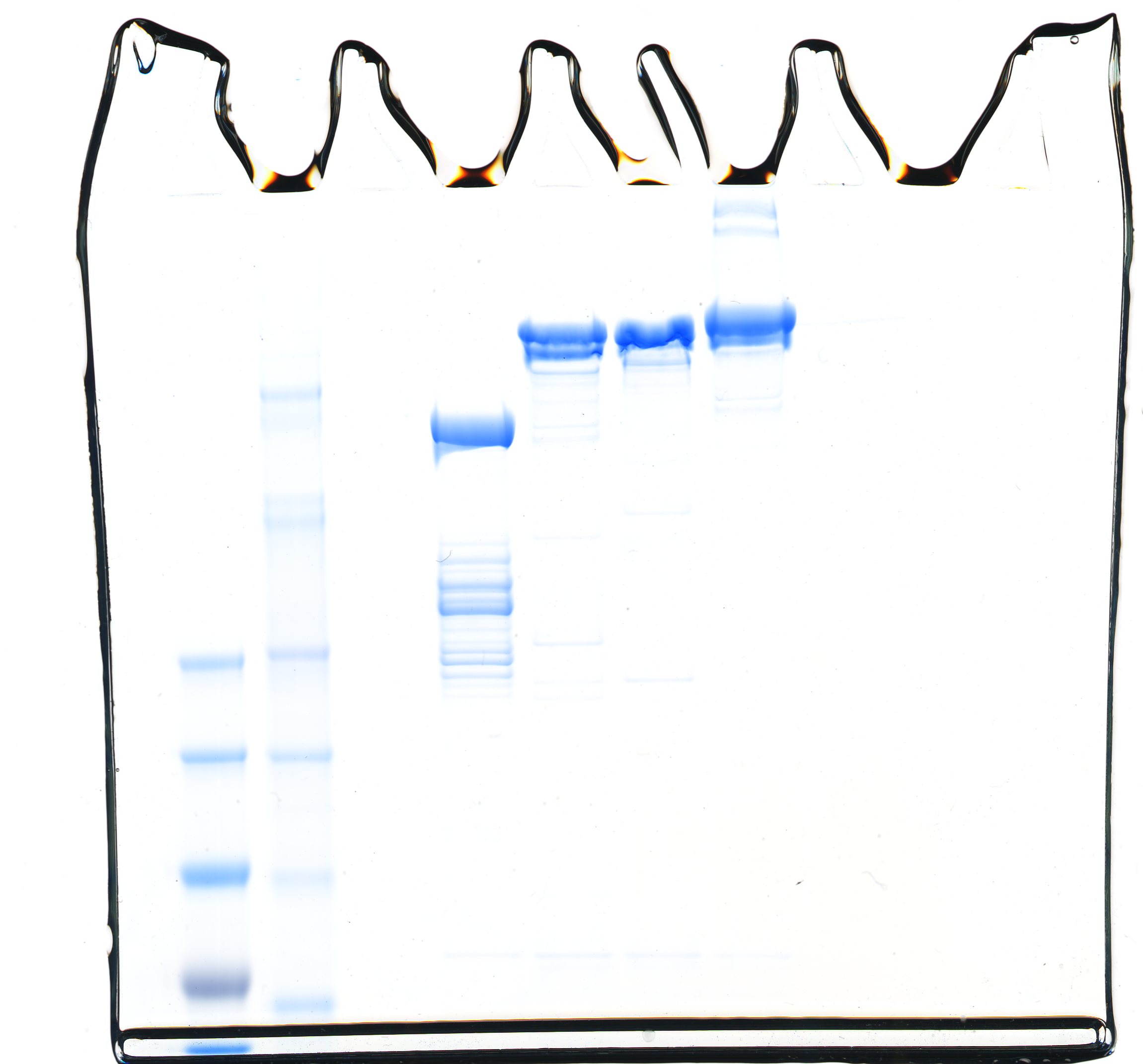

Supplement: Source data 1. [file elife-80310-data1.zip › Figure 1C - source data.jpg]

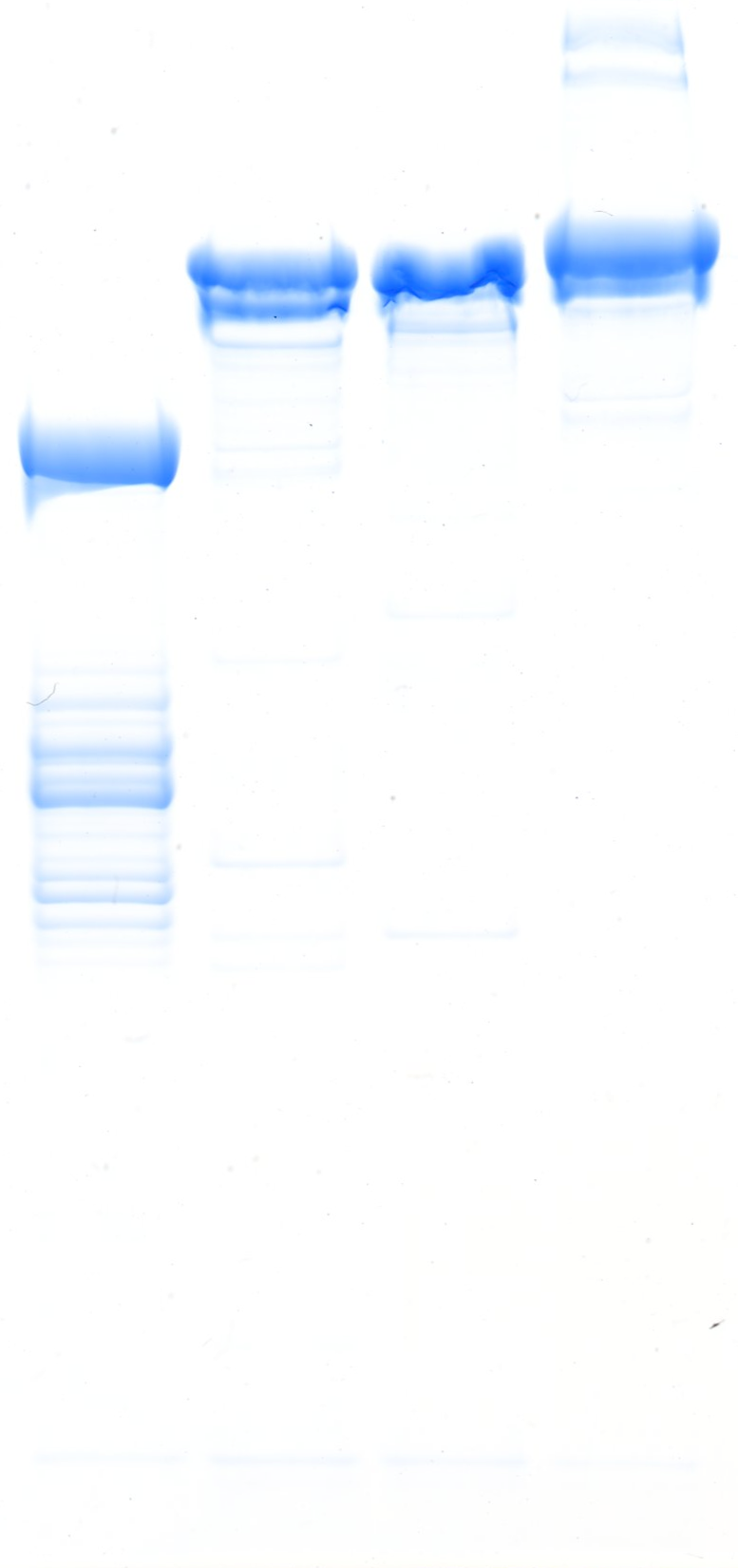

Supplement: Source data 1. [file elife-80310-data1.zip › Figure 1C - source data cropped.png]

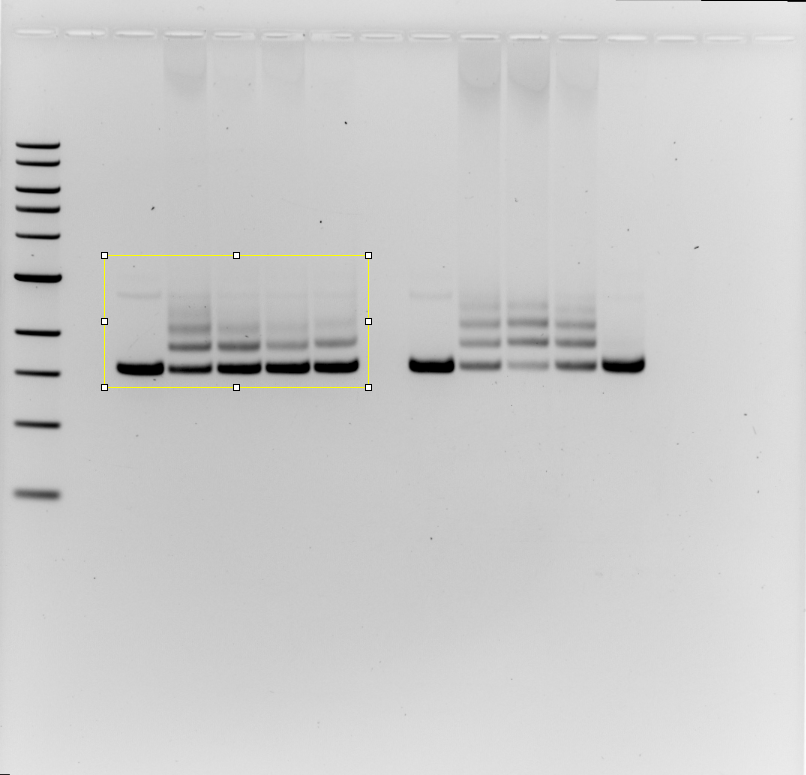

Supplement: Source data 1. [file elife-80310-data1.zip › Figure 2C - source data cropped.png]

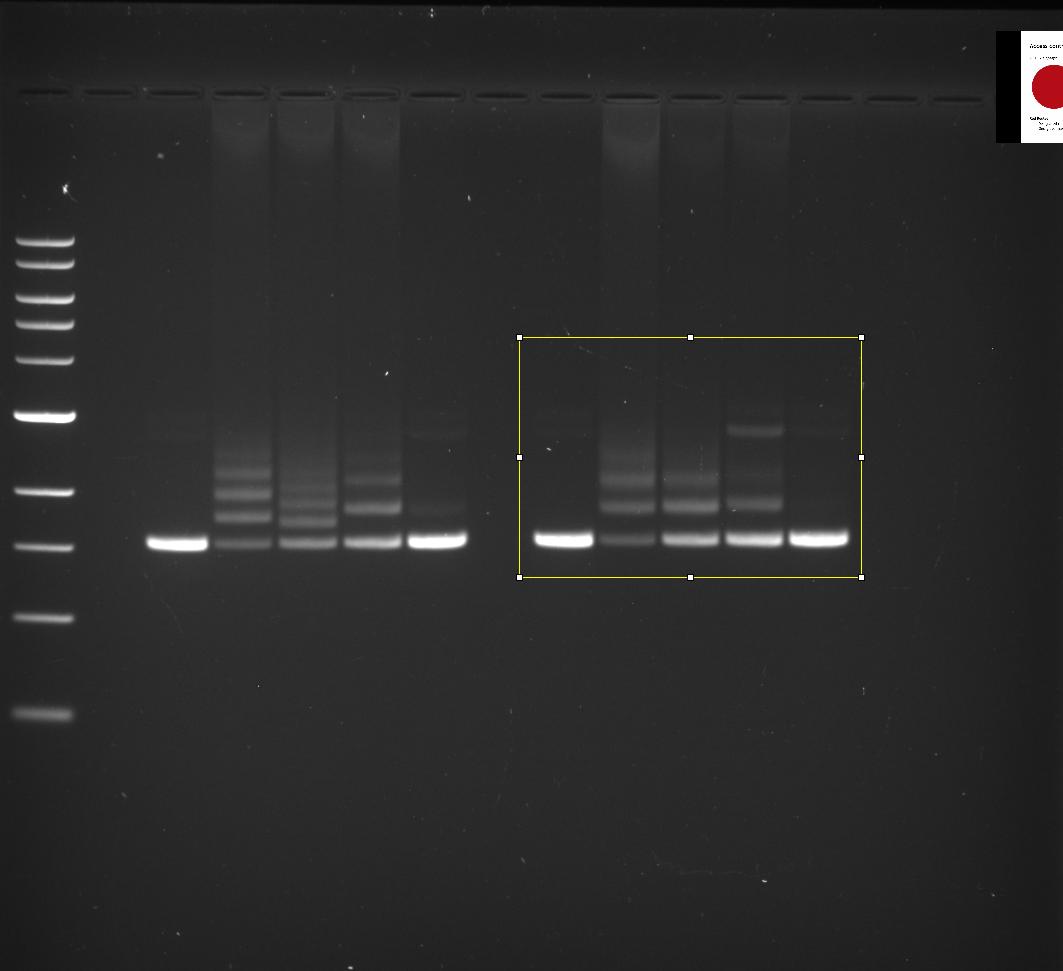

Supplement: Source data 1. [file elife-80310-data1.zip › Figure 2D - source data cropped.png]

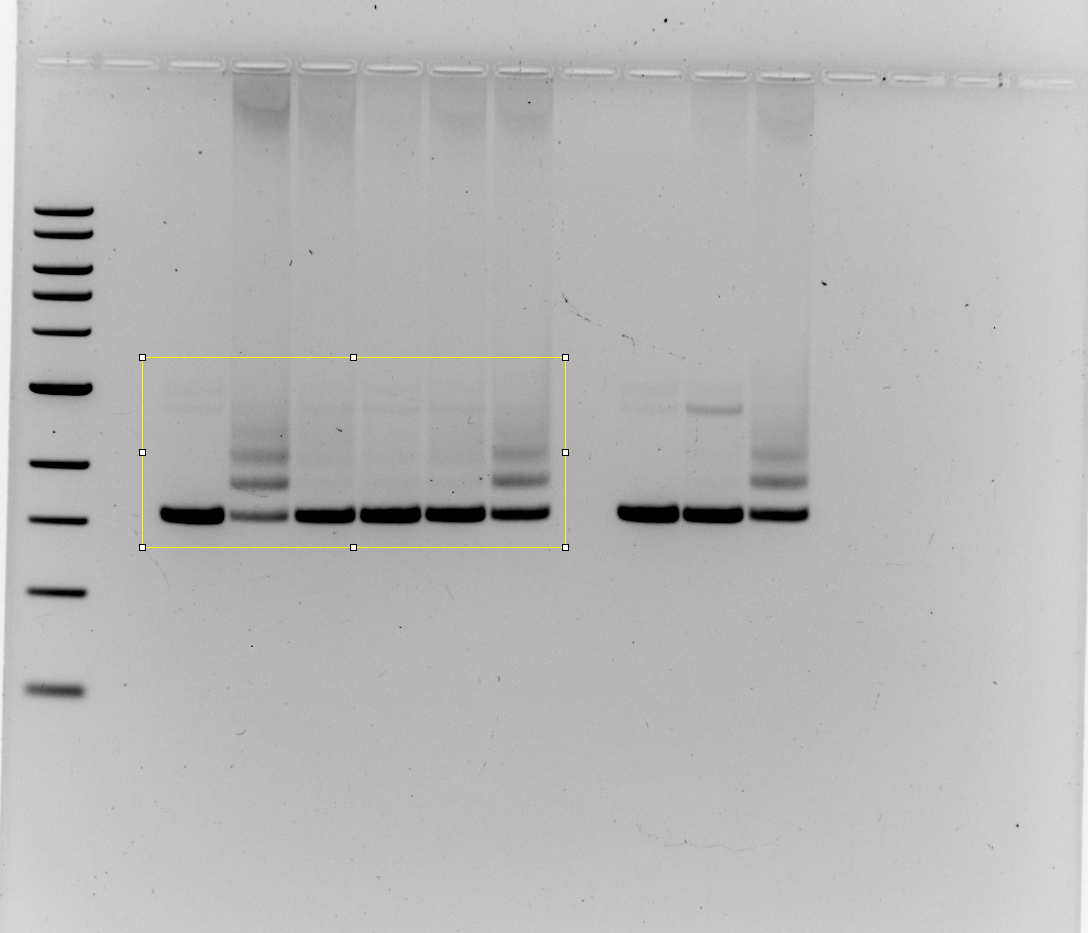

Supplement: Source data 1. [file elife-80310-data1.zip › Figure 2E - source data cropped.png]

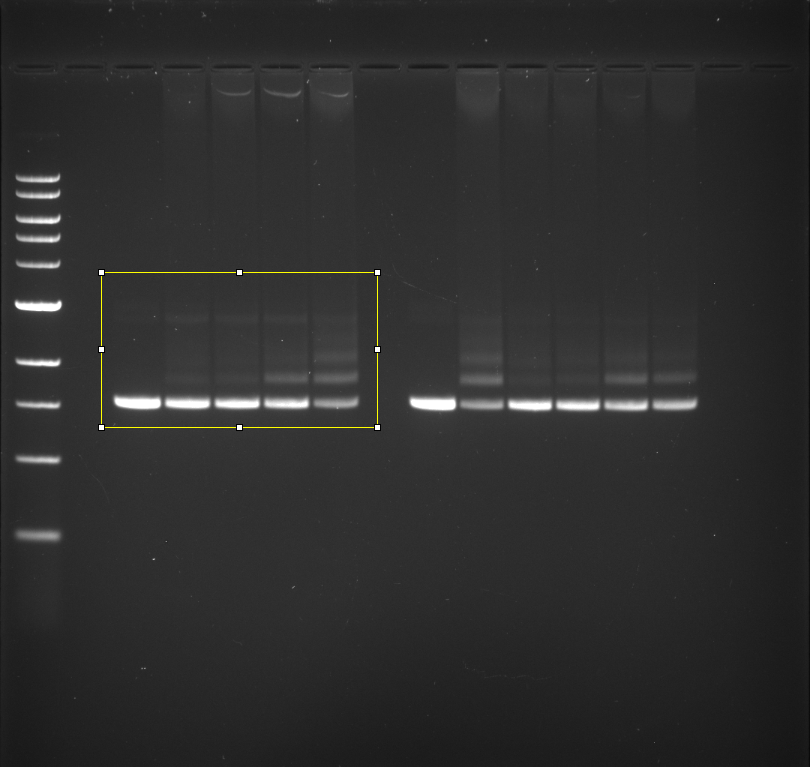

Supplement: Source data 1. [file elife-80310-data1.zip › Figure 2 - figure supplement 1A - source data cropped.png]

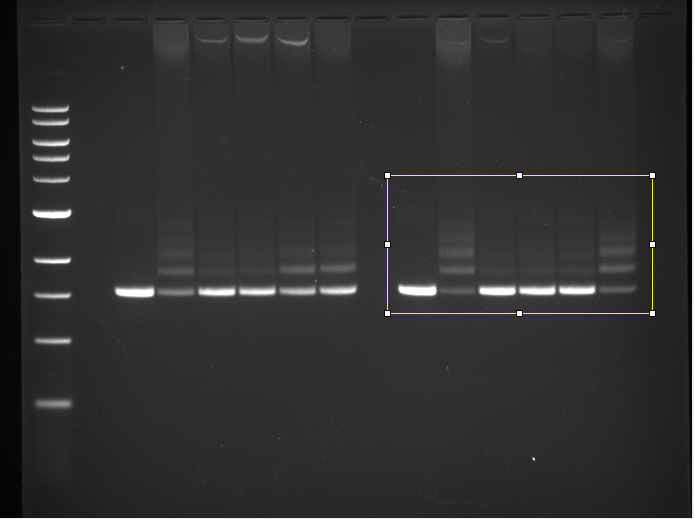

Supplement: Source data 1. [file elife-80310-data1.zip › Figure 2F - source data croppped.png]

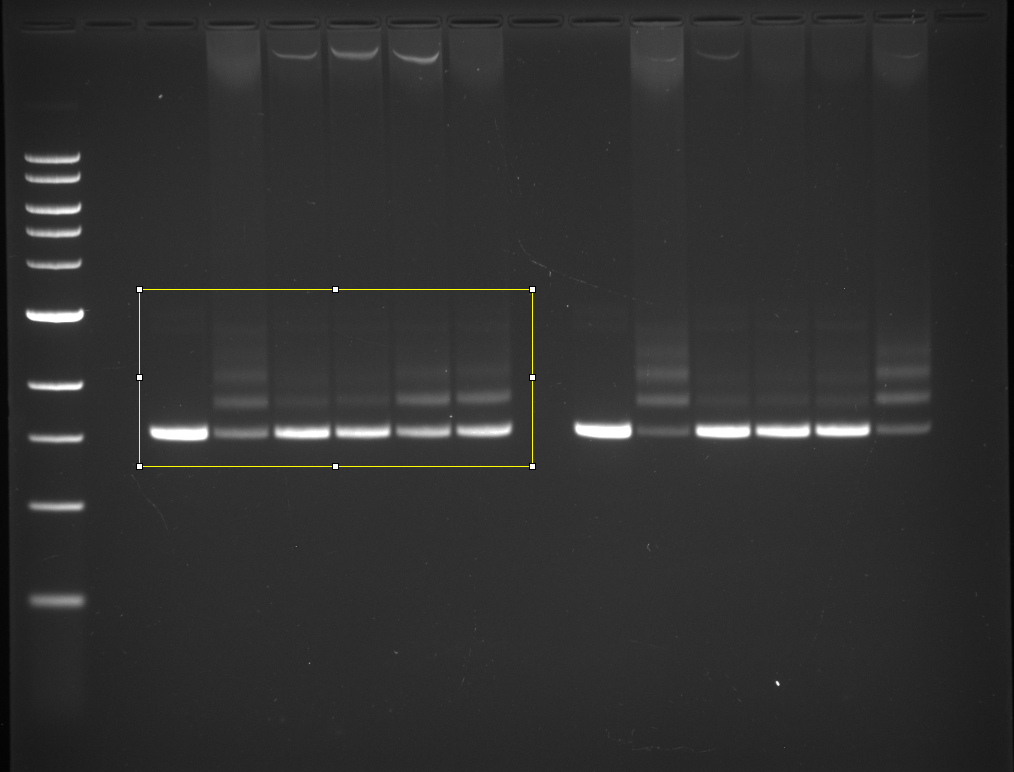

Supplement: Source data 1. [file elife-80310-data1.zip › Figure 2G - source data cropped.png]

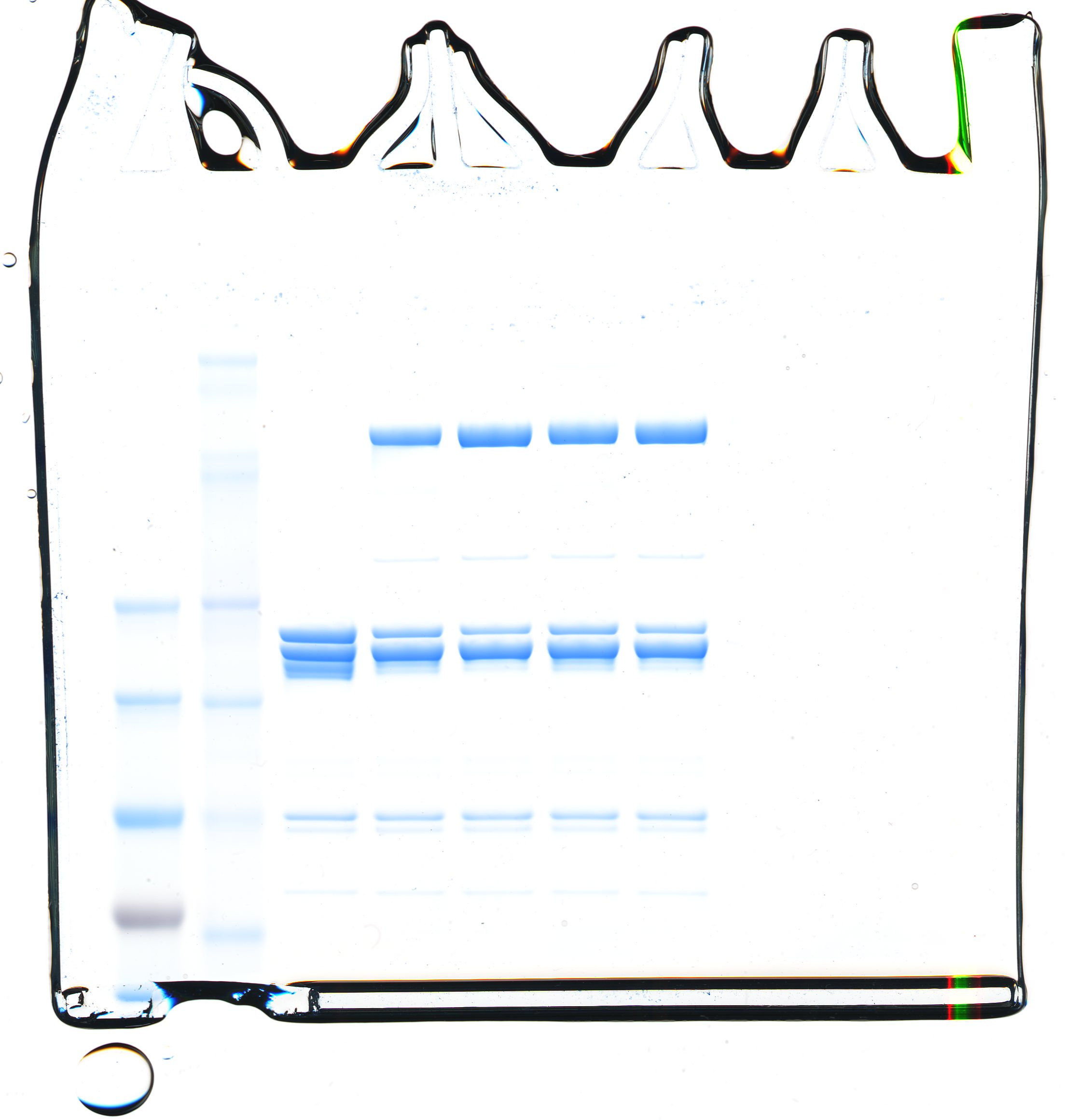

Supplement: Source data 1. [file elife-80310-data1.zip › Figure 3A - source data.jpg]

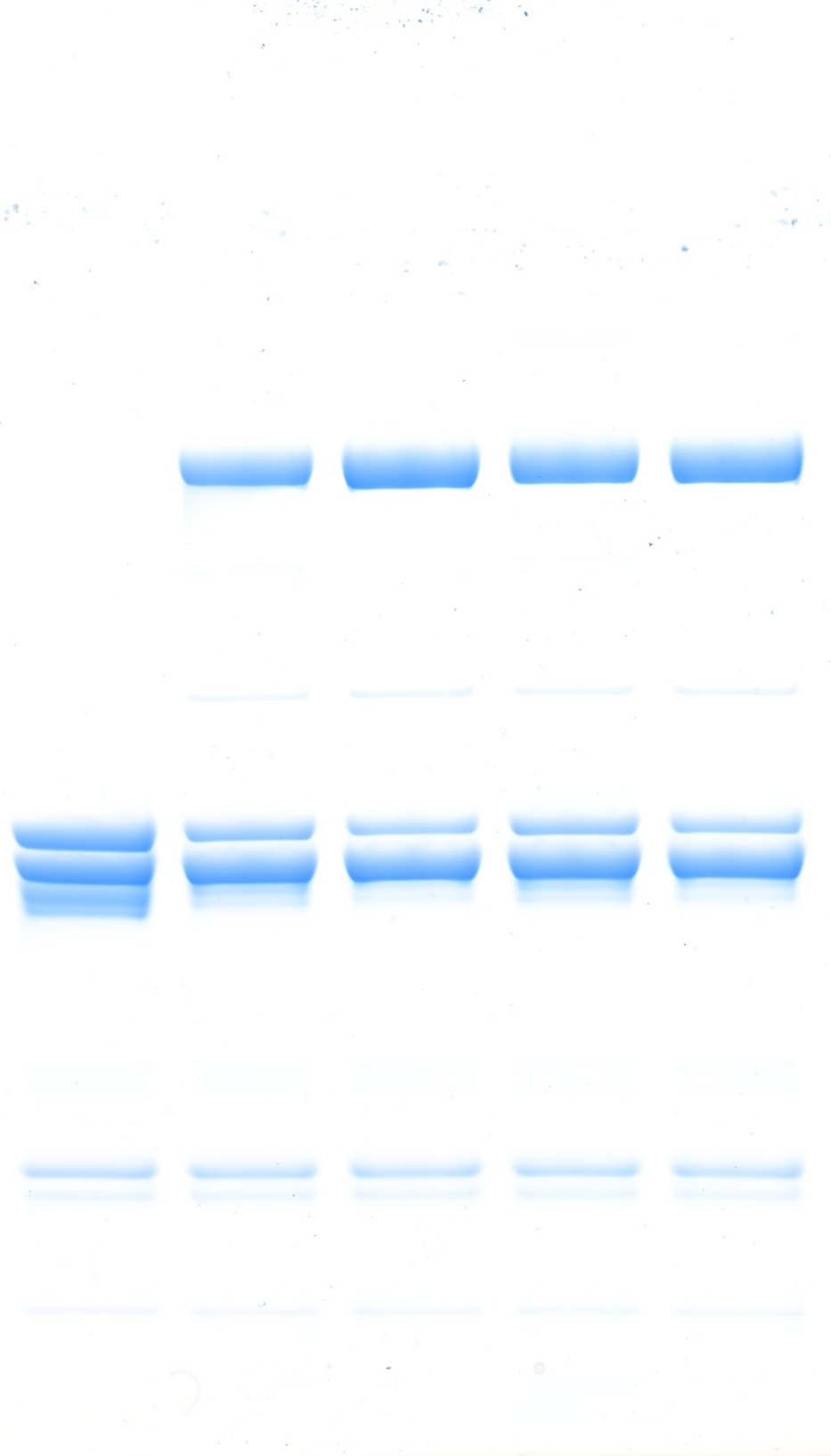

Supplement: Source data 1. [file elife-80310-data1.zip › Figure 3A - source data cropped.png]

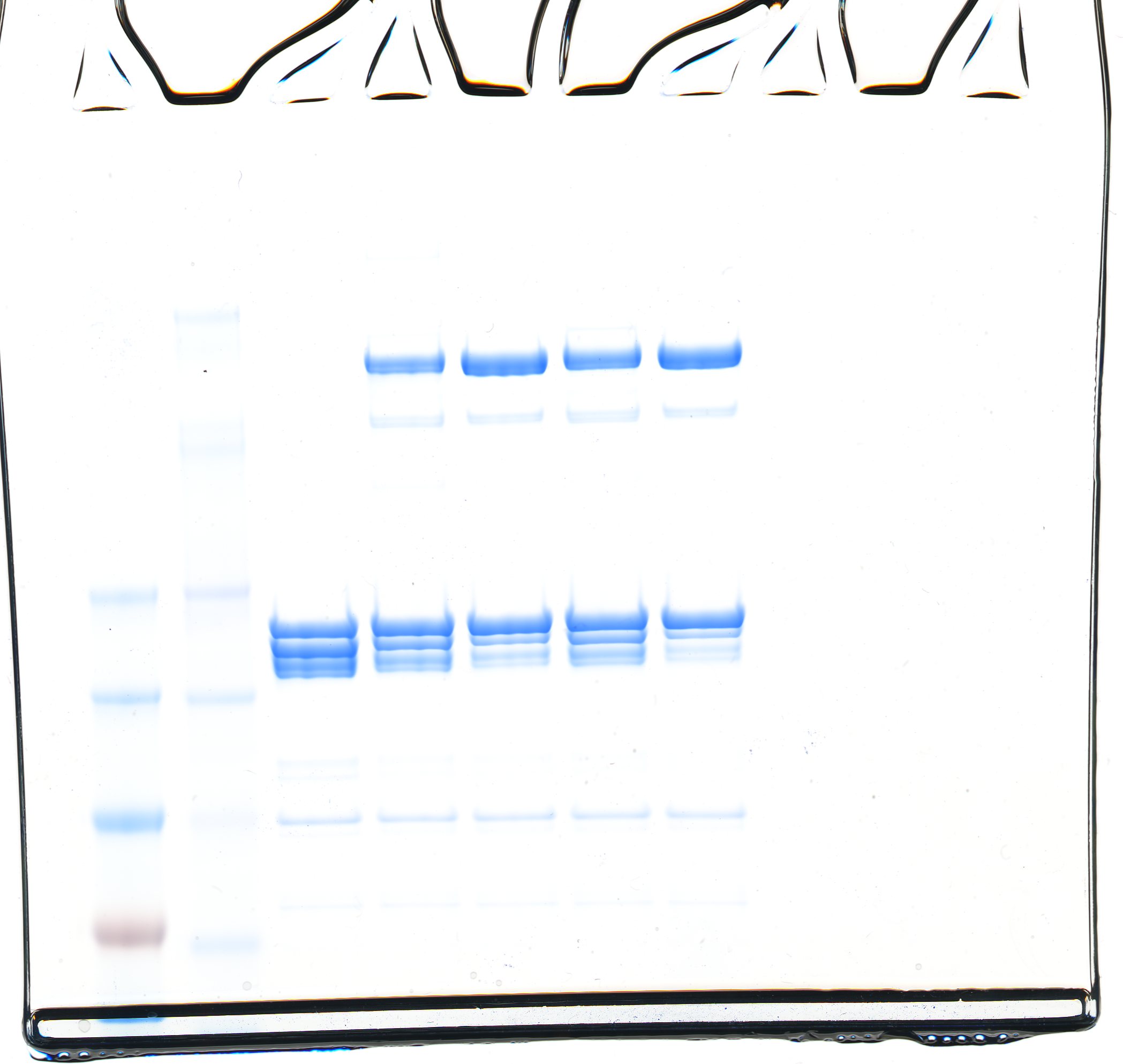

Supplement: Source data 1. [file elife-80310-data1.zip › Figure 3B - source data.jpg]

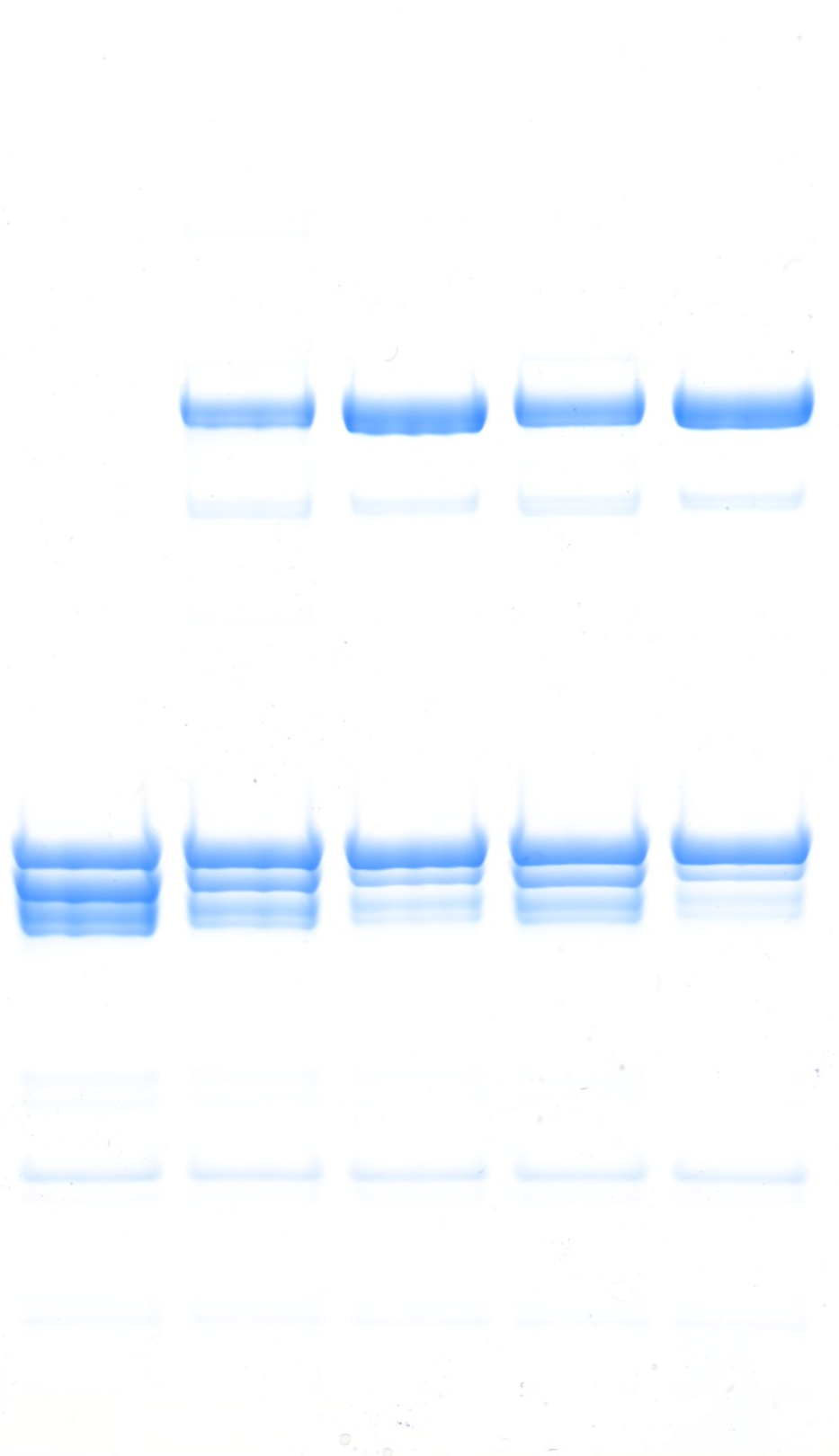

Supplement: Source data 1. [file elife-80310-data1.zip › Figure 3B - source data cropped.png]

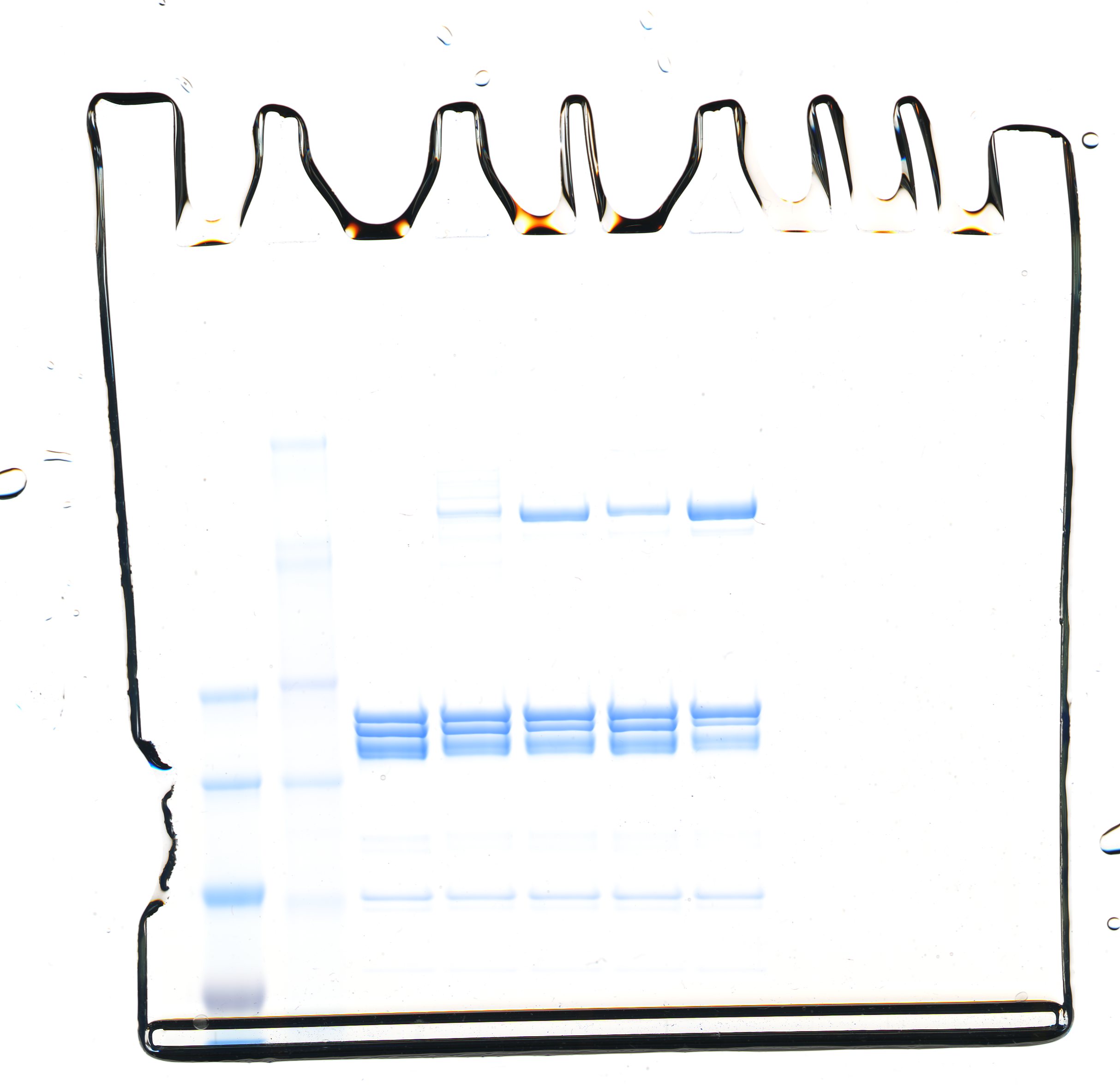

Supplement: Source data 1. [file elife-80310-data1.zip › Figure 3C - source data.jpg]

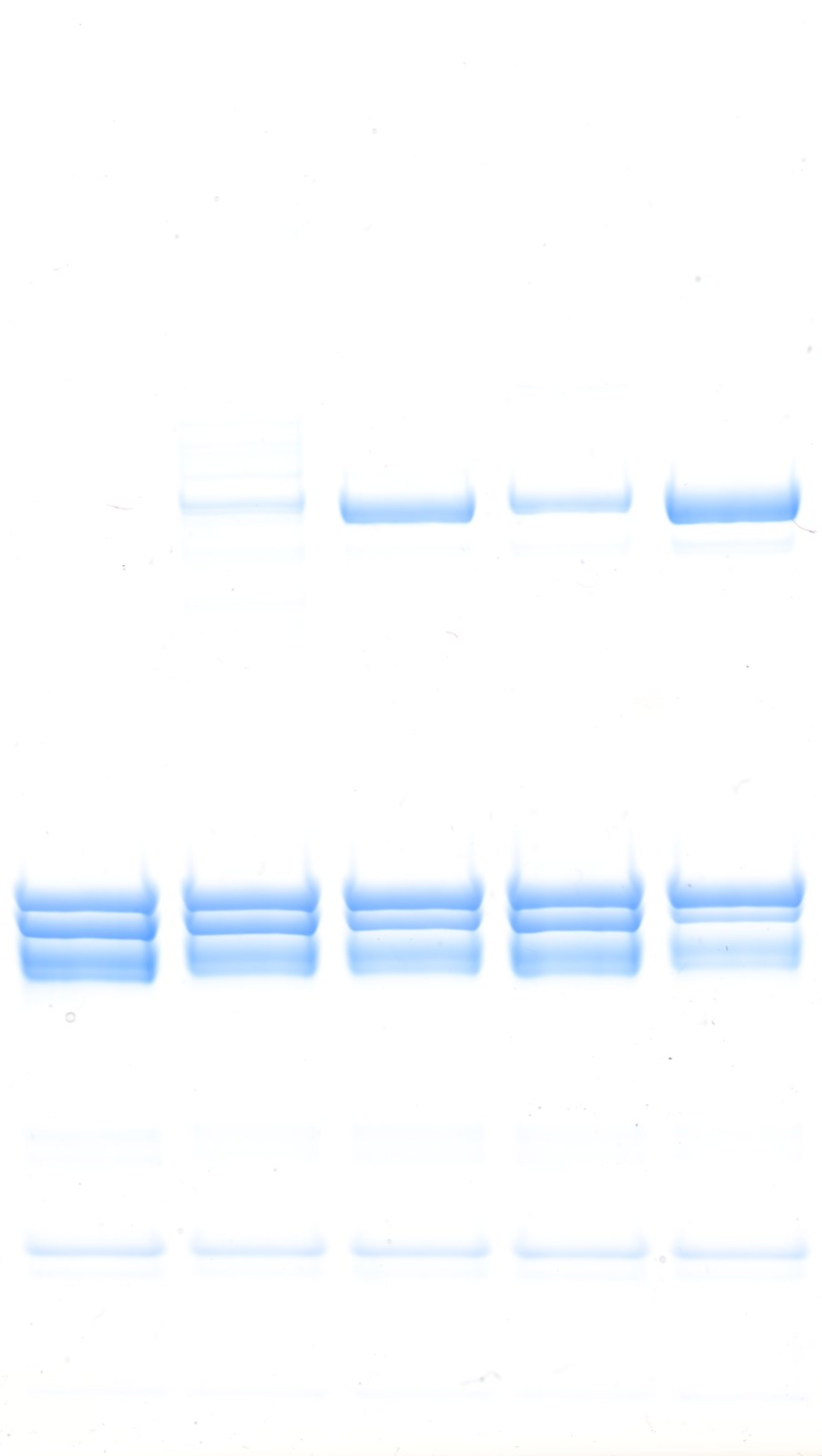

Supplement: Source data 1. [file elife-80310-data1.zip › Figure 3C - source data cropped.png]

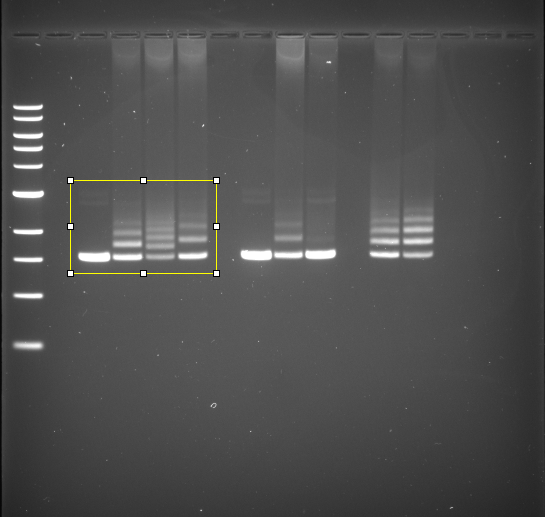

Supplement: Source data 1. [file elife-80310-data1.zip › Figure 3E - source data cropped.png]

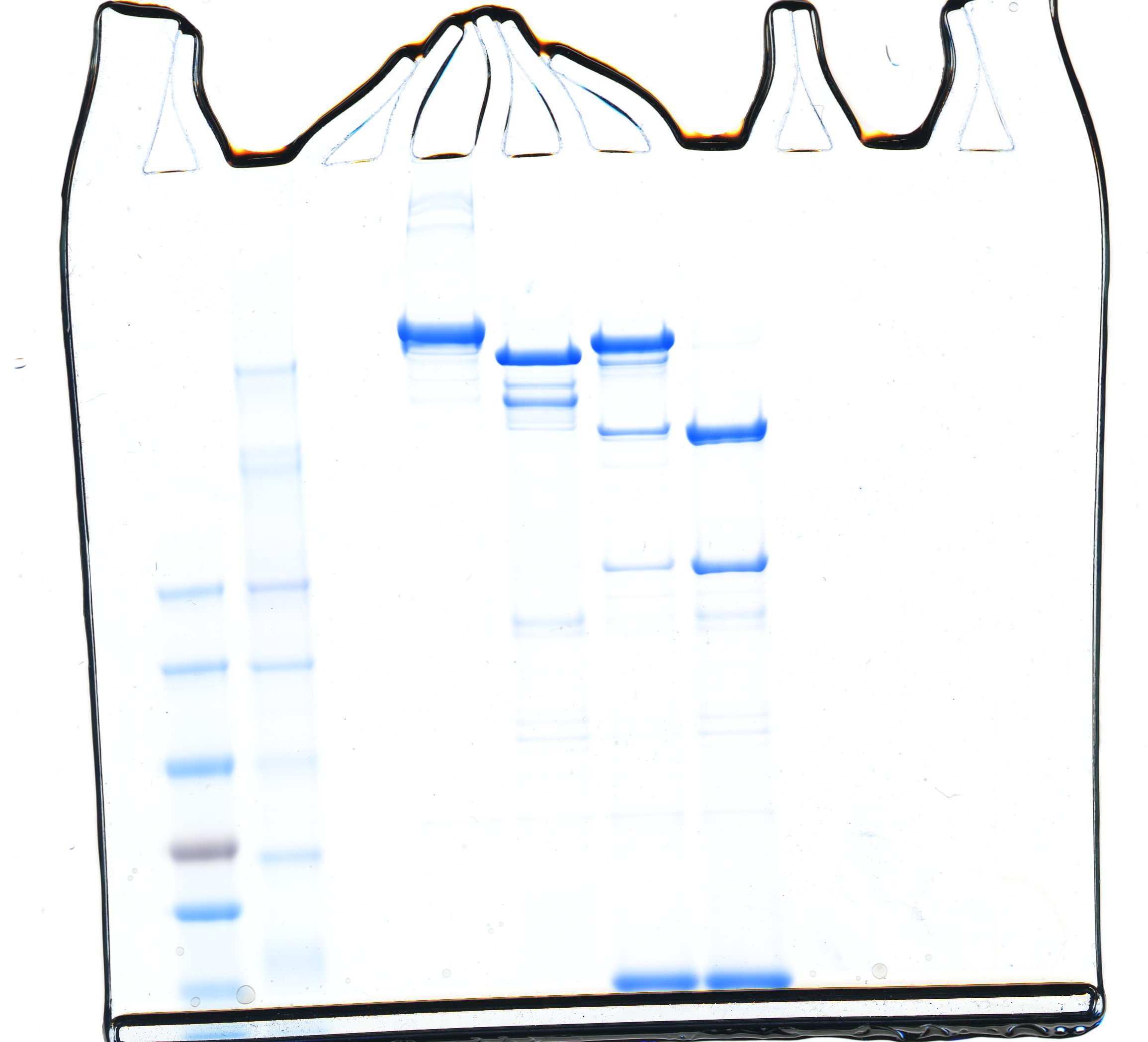

Supplement: Source data 1. [file elife-80310-data1.zip › Figure 3 - figure supplement 2C - source data.jpg]

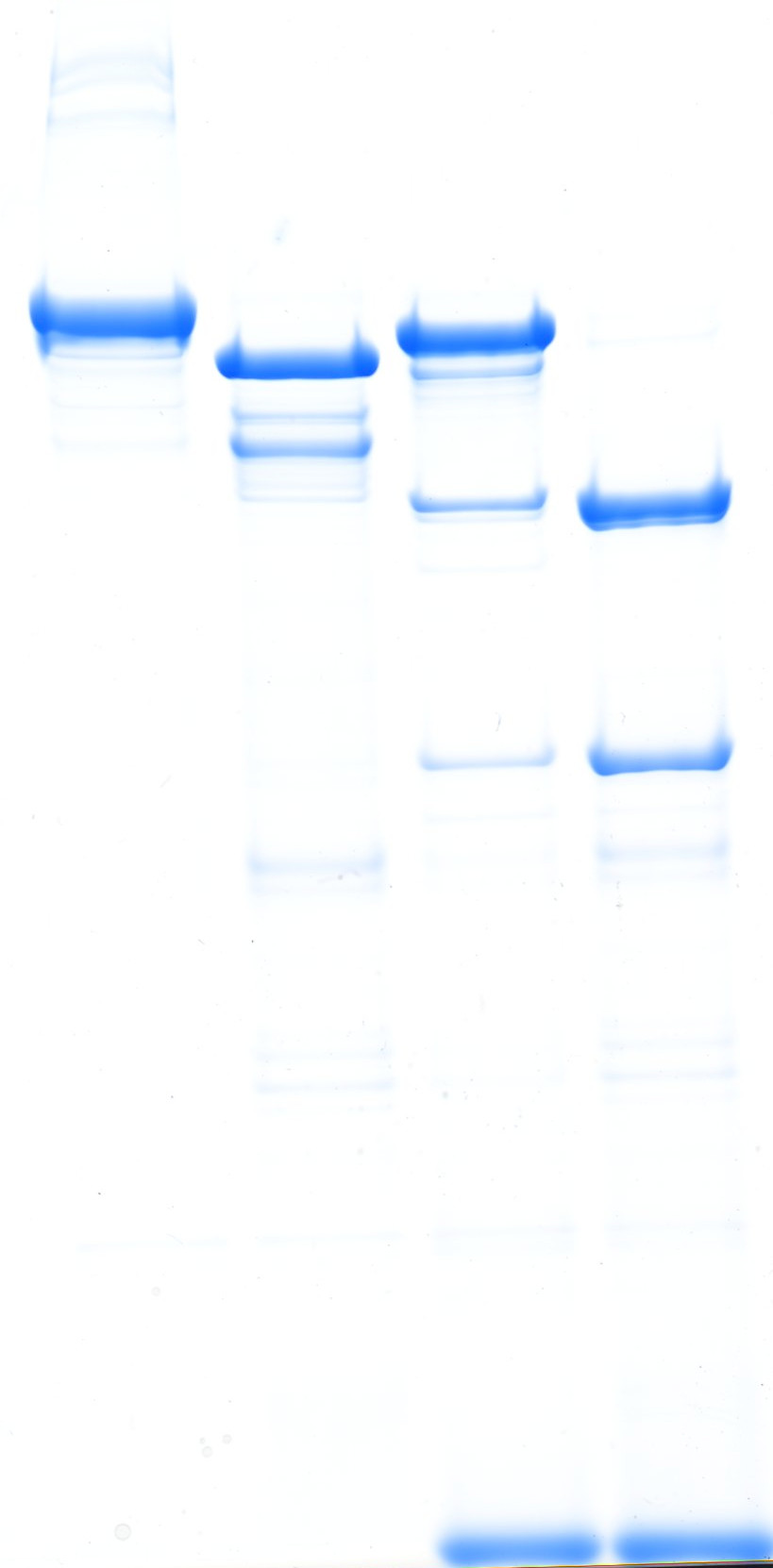

Supplement: Source data 1. [file elife-80310-data1.zip › Figure 3 - figure supplement 2C - source data cropped.jpg]

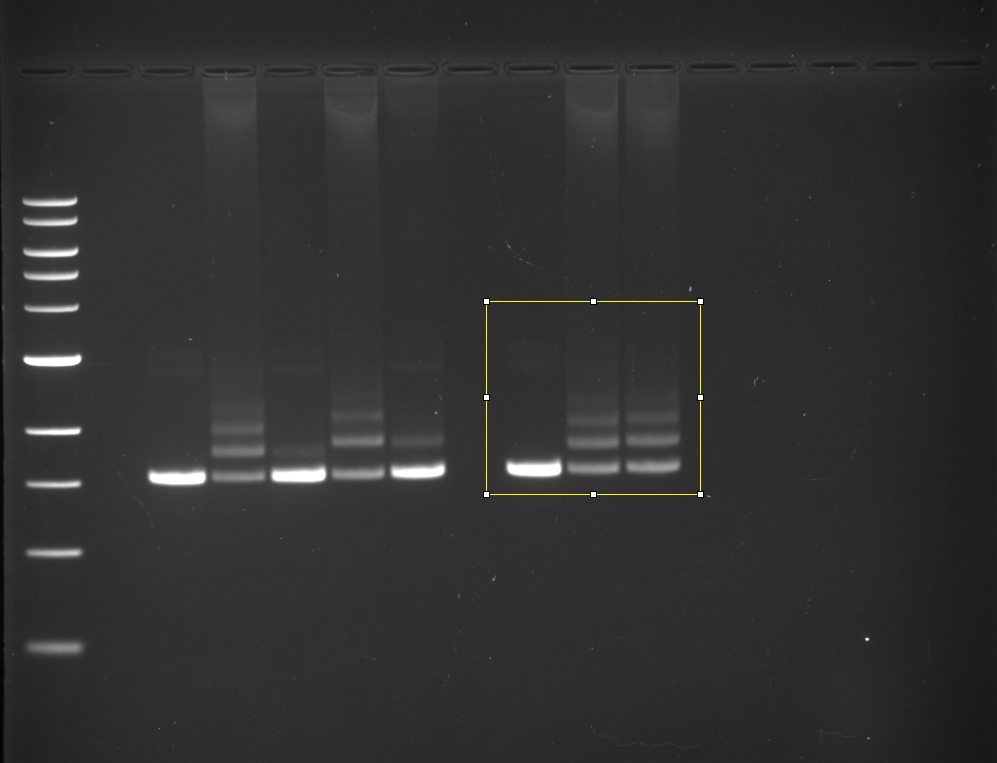

Supplement: Source data 1. [file elife-80310-data1.zip › Figure 3F - source data cropped.png]

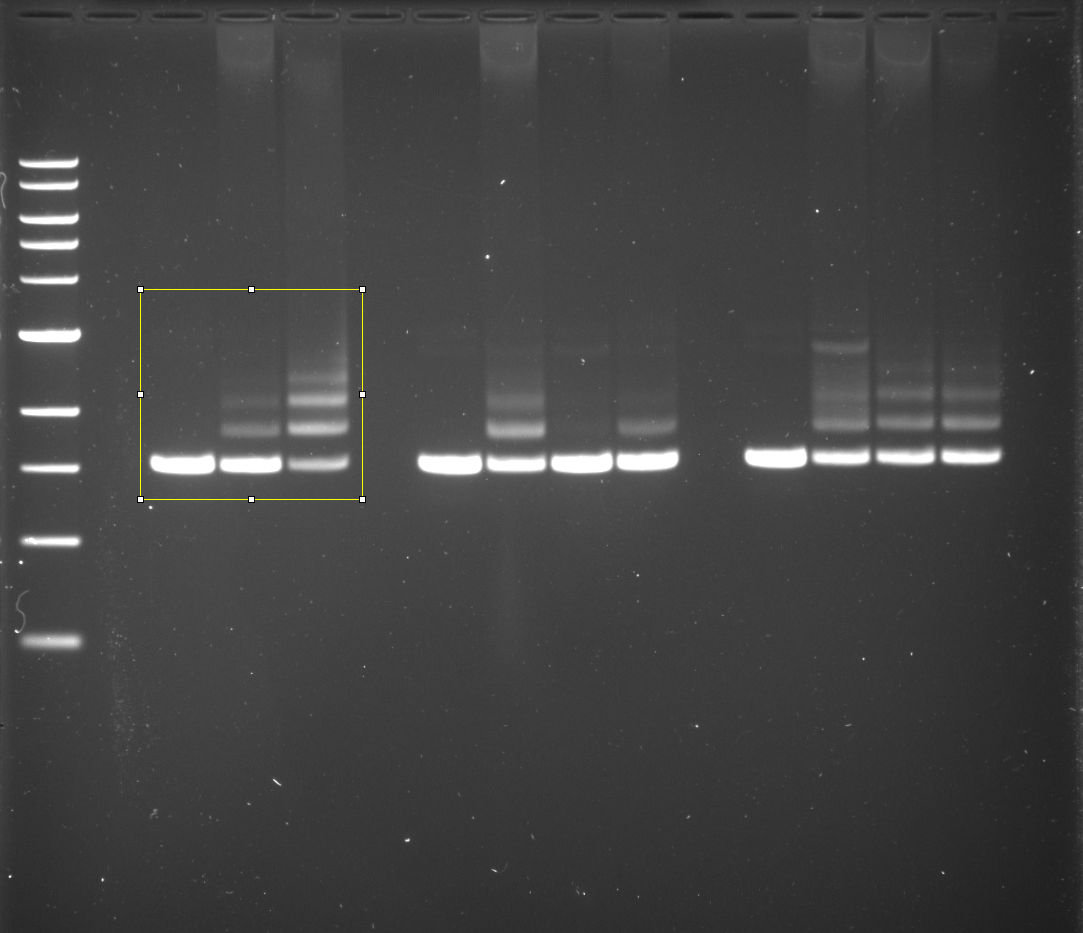

Supplement: Source data 1. [file elife-80310-data1.zip › Figure 3G - source data cropped.png]

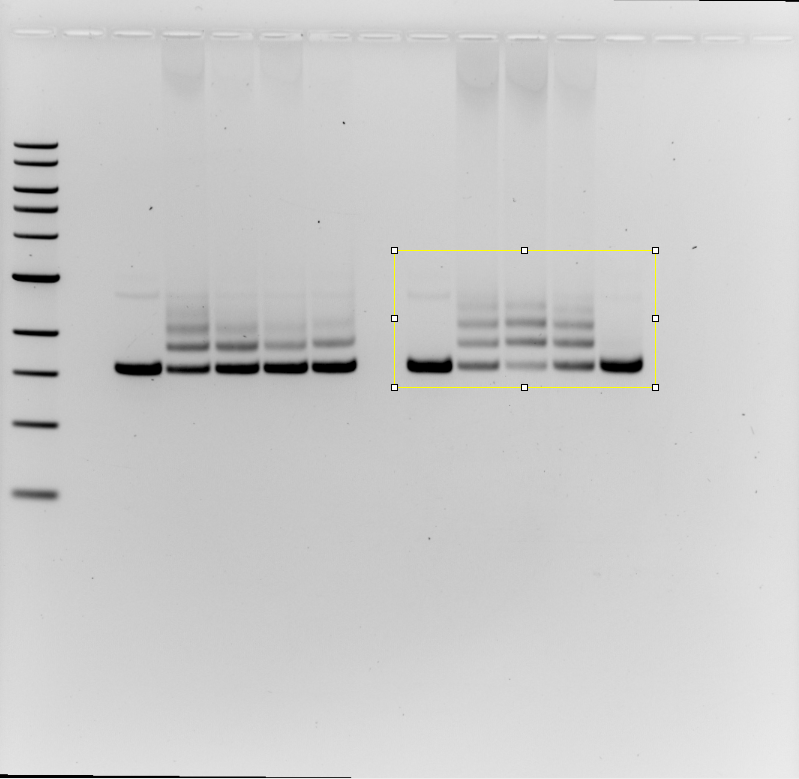

Supplement: Source data 1. [file elife-80310-data1.zip › Figure 3H - source data cropped.png]
